# Casebooks in Early Modern England: Medicine, Astrology, and Written Records

**DOI:** 10.1353/bhm.2014.0066

**Published:** 2014

**Authors:** Lauren Kassell

**Keywords:** casebooks, medical records, astrology, paper technologies, cases, patients, Simon Forman, Richard Napier

## Abstract

Casebooks are the richest sources that we have for encounters between early modern medical practitioners and their patients. This article compares astrological and medical records across two centuries, focused on England, and charts developments in the ways in which practitioners kept records and reflected on their practices. Astrologers had a long history of working from particular moments, stellar configurations, and events to general rules. These practices required systematic notation. Physicians increasingly modeled themselves on Hippocrates, recording details of cases as the basis for reasoned expositions of the histories of disease. Medical records, as other scholars have demonstrated, shaped the production of medical knowledge. Instead, this article focuses on the nature of casebooks as artifacts of the medical encounter. It establishes that casebooks were serial records of practice, akin to diaries, testimonials, and registers; identifies extant English casebooks and the practices that led to their production and preservation; and concludes that the processes of writing, ordering, and preserving medical records are as important for understanding the medical encounter as the records themselves.

The astrologer sits in his consulting room “Before a Square Table, covered with a greene Carpet, on which lay a huge Booke in *Folio*, wide open, full of strange Characters, such as the *Ægyptians* and *Chaldaens* were never guiltie of.” The room is furnished “with all the superstitious or rather fayned Instruments of his cousening Art.” This is John Melton’s 1621 caricature of an astrologer. To find him, he explains, you followed a gaggle of women down the backstreets of London to his house. When you arrive, you can ask, for a price, the whereabouts of lost or stolen goods, how many children you will have, or the cause of your disease. In exchange for money the astrologer will give you many words of little value. Melton presents the astrologer as a fraud who trades in fake learning and empty results.^1^

This article is about the book on the astrologer’s table. This is a casebook. Casebooks are all that survives from early modern encounters between medical practitioners and their patients. Some were produced by astrologers, others by physicians. They derive from a range of intellectual traditions, and they share a common purpose: to document medical encounters. Historians have used casebooks to study experiences of illness and healing, while exercising caution in reading them as direct representations of the patients’ perspective and supplementing them with letters, diaries and other ego documents.^2^ This article is the first study to take [Other P-596] casebooks, as material and intellectual artifacts of medical practice, as its subject. It raises questions about how many early modern practitioners wrote casebooks, and how and why they kept them. It also situates these records within broader trends in “paper technologies,” scholarly practices, and the production of medical knowledge.^3^ Finally, it identifies extant casebooks and related documents and provides a framework for modern scholars to use them critically.

“Casebook” is a collective term for a variety of records of practice, mostly generated by literate men, unpolished, not for the ready use of an intended reader. Like other sorts of early modern life writing, they often take the form of lists rather than narratives.^4^ Some are modeled on diaries, recording day-to-day practice, and shaped by mercantile habits of account keeping. Others draw on scholarly conventions of note taking and commonplacing.^5^ Whether kept by astrologers, physicians, [Other P-597] apothecaries, surgeons, or others, or written during the consultation or after the fact, casebooks were material products of the early modern medical encounter. They need to be considered as artifacts of and instruments in healing dynamics.

## Simon Forman’s and Richard Napier’s Casebooks

One of the astrologers mentioned by Melton is Simon Forman, probably the most popular astrologer in Elizabethan London. Forman quarreled with the London College of Physicians, and his reputation included equal measures of quackery, demonic magic, and womanizing. His casebooks for 1596–1603 record roughly ten thousand consultations, mostly in his hand, occasionally written by an assistant. Forman’s practices were continued by Richard Napier, a Buckinghamshire clergyman who became Forman’s astrological protégé in the late 1590s. Napier kept casebooks from 1597 until his death in 1634, written by both himself and assistants. Together Forman’s and Napier’s casebooks fill fourteen thousand pages and document eighty thousand consultations. This is the largest set of records of its kind, and Napier’s is the only complete surviving set of early modern casebooks. The richness of this material is matched by its inaccessibility. They are astrological records written in rushed handwriting and cryptic notation.^6^

Most of Forman’s and Napier’s records are for horary interrogations, calculations based on the positions of the stars when a question was asked. The astrologer recorded the client’s name and age, whether the client appeared in person or sent a message, the question posed, and always the moment at which the consultation began or the message arrived. Drawing [Other P-598] a figure, or chart, mapping the positions of the stars for that moment, the astrologer recorded his judgment and, in some cases, a prediction, remedy, recommended course of action, payment, or outcome. Prescriptions, payments, and consultations to which the astrologer traveled were recorded in separate notebooks. Astrology provided a formula for recording systematic records.

Analyses of Forman’s and Napier’s casebooks have centered on the popularity of astrologers, especially with women.^7^ Melton and twentieth-century historians, notably Keith Thomas and Michael MacDonald, ask why so many people considered the astrologers’ art to be credible and argue it was a solace for the anxieties of everyday life in early modern England.^8^ Such explanations rely on functional arguments and risk reducing the medical encounter to a pristine transaction between two calculating individuals. As I have proposed elsewhere, a more fruitful set of questions might focus on why the astrologers recorded systematic information and what these records reveal about the dynamics of healing. Horary astrology was integral to Forman’s and Napier’s procedures and casebooks embody this: as material objects they were present in the consultation. The patient did not necessarily believe in astrology; rather, the astrologer had to establish his authority and to negotiate a judgment about the nature of the disease and the possible therapies for it. Gender, moreover, was a defining feature of Forman’s practice, not because women were more ill, but because their health was tied to their reproductive capacity. This made astrology, according to Forman, particularly necessary in dealing with women’s health. The astrologer could discern his patients’ often-concealed sexual activities and emotional preoccupations. The astrologer and his patient negotiated an exchange of trust for true judgments. Casebooks were central to this dynamic. They were instruments for judging the causes of disease, status objects demonstrating the astrologer’s expertise, and records of these practices. With pen and paper, the astrologer located each patient within the cosmos and signaled his authority to do so.^9^[Other P-599]

This analysis risks fetishizing the astrologer’s pen, making it the defining tool in encounters between vulnerable women and potent men. My work to identify other English casebooks began to assess the representativeness of Forman’s and Napier’s records, in terms of the numbers of clients and the proportion of women among them. It soon became clear that before we could answer these questions, we needed a better understanding of the history of medical record keeping in early modern England.

## What Is a Casebook?

To identify casebooks, I also needed to define them. The term “casebook” seems to date from the late seventeenth century, when it was used in a legal context; it was applied to medicine only in the middle of the eighteenth century.^10^ Casebooks are serial records of practice. When written by physicians, surgeons and apothecaries, they contain records of medical consultations. Astrological casebooks are related, but distinct. They record medical questions alongside consultations about, for instance, marital fortune and the identity of a thief. Many practitioners did not label their collections, but some called them various sorts of books (“bosom book,” “book of judgments,” “book of remedies”), “journals,” “experiments and cures,” or, in the Latin tradition, “cures,” “cases,” “diaries,” “histories,” or “observations.” The term “casebook” usefully indicates a body of manuscripts that record a series of consultations, but these documents do not constitute a prescribed genre uniform in mode of production, content, or epistemology.

The richest surviving casebooks—by Joseph Binns, Theodore de Mayerne, John Symcotts, and Thomas Willis—are highlighted by other scholars. The Sloane Collection contains many more, catalogued as medical observations, cases, case notes, and diaries. I trawled manuscripts classified as medical and astrological notes, limiting my study, with one eye on Forman and Napier, to England and stopping in 1700. After this date medical records became more common and formulaic.^11^ These papers were inconsistently or often erroneously catalogued, and survival rates were poor. I identified thirty-six sets of medical and eleven sets of astrological casebooks. This represents the work of a small fraction of the [Other P-600] English practitioners circa 1450 to 1700. We do not know how many others wrote casebooks. Some may have recorded particular cases of note, others a full series of their daily practices. We know, for instance, that William Drage, an apothecary–physician known for his medical work on witchcraft, recorded fourteen hundred medical cases from 1658 to at least 1664. These are lost.^12^ Where the miscellaneous papers of practitioners do survive, such as those of Philip Moore, who recorded remedies and copies of alchemical works in Northampton in the 1570s,^13^ and the better-known collections of William Butler (1535–1618), the Cambridge physician with elite contacts, we do not know whether they recorded casebooks that are now missing.^14^ The surviving examples are the product of the practices of collecting and preserving records as much as of producing them.^15^

While the surviving astrological casebooks follow a format much like Forman’s and Napier’s, the medical casebooks are more diverse. Some were written by unknown practitioners, others record only a few dozen cases. The intractability of these papers prompted me to see them as artifacts of medical practice as much as records of once meaningful information. Combined with evidence for medical record-keeping habits in printed medical works, and reflexive comments by practitioners about their practices, it is possible to identify some basic trends across the period.

Before summarizing these trends, it must be noted that casebooks, by definition, were written by literate practitioners. Some had received more formal education. Roughly half are in Latin, half in English, with some using both languages. This project encompasses literate practitioners; it is not restricted to learned physicians. Only one woman appears among the scores of practitioners discussed in this article.

Within learned medicine, the case had been a recognized problem since antiquity. In the fourteenth and fifteenth centuries European medical writings increasingly detailed narratives of particular cases and particular cures, especially if they were unusual in some way.^16^ Recent scholarship has charted shifts from the medieval *experimentum* and *curatio* to the rise of observation as an epistemic genre in the sixteenth century; cases demonstrating a physician’s successful cures were replaced by case [Other P-601] histories centering on the patient and his disease.^17^ Astronomers instituted sustained regimes of observation in the late fifteenth century, fueling a growing interest in particulars and the practices of observation across various disciplines, including medicine.^18^ In the sixteenth century, attention to medical cases was also prompted by the recovery of ancient medical texts, especially Galen’s analyses of his cases and the recently translated Hippocratic *Epidemics*.^19^ In 1573, Francois Valleriola, who held the chair of medicine in Turin, wrote, “[Hippocrates] wrote on tablets all that he saw occurring in the sick person, and narrated the complete *historia* of the disease and what happened to the sick each day, each hour, each moment, giving specifically the name of each person.… ” Modeling his practices on Hippocrates, he then “reworked for general use the things I wrote down, taking into considerations only those diseases that appeared to me most dangerous and of dubious treatment.”^20^ Here we see two stages of writing: the doctor wrote initial notes, and subsequently reworked exceptional cases for his readers. The second stage has interested historians of knowledge; the first stage documents the role of writing, either at the time or from memory later in the day, in the medical encounter. The processes of producing medical records are the subject of this article. To understand these processes, we need to study their products.

Practices of recording medical records—patient’s name, date, place, recipes and remedies used, and outcome of the case—had spread among [Other P-602] learned physicians in the second half of the sixteenth century, beginning in the Italian universities circa 1550, then advocated by the Parisian Hippocratics in the 1570s.^21^ Attention to the epistemic implications of medical observations was codified in Baconian experimentation and the culture of fact in the middle of the seventeenth century. Francis Bacon had echoed earlier scholars when he lamented that physicians had lost “the ancient and serious diligence of Hippocrates, which used to set down a narrative of the special cases of his patients, and how they proceeded, and how they were judged by recovery or death.”^22^ This story culminates in the histories of diseases written by Thomas Sydenham, known as the English Hippocrates, from the 1660s, typically understood as marking a shift in the object of medical inquiry from the patient to the disease.^23^ Alongside and contributing to these epistemic shifts, the record-keeping habits of doctors changed: they borrowed scholarly methods and paper technologies from state administration and financial accounting to record details of their practices. They were trained, for instance, to collect commonplaces, not cases.^24^

In the English context, these narratives could be expanded to encompass the wrangling over medical politics in the 1650s and 1660s. Proponents of Helmontian medicine drew a lineage between Hippocratic record keeping and their methods. After the Restoration, medical records featured in the efforts of the College of Physicians to reinvent itself as a learned society. Through the 1660s some of its members gathered in regular juntos focused on “histories of diseases” from their own practices and other remarkable cases.^25^ It was also proposed that hospital physicians should “keep exact accounts” of their cases, successful and not, and that these should be registered in the college archives.^26^

This attention to the production of medical knowledge ensures that doctors are included within historical epistemology, yet misses an opportunity to use the “materiality of medical writing” to revisit questions about [Other P-603] medical encounters and the creation of the medical subject.^27^ These are old themes in the history of medicine. In 1976, building on Erwin Ackerknecht’s writings about what doctors do, Nicholas Jewson schematized the modes of production of medical knowledge, positing the “disappearance of the sick man” with the shift from bedside to hospital to laboratory medicine circa 1770 to 1870. For Jewson, bedside medicine perceived the sick man as a person, hospital medicine as a “case,” and laboratory medicine as a cell complex. In bedside medicine, the patient’s narrative mattered; “diagnosis was founded upon extrapolation from the patient’s self report of the course of his illness.”^28^ The form of the encounter between a patient and a practitioner shaped the medical subject. Jewson, as far as I am aware, did not reflect explicitly on the nature of the medical record.

For Jewson, physicians practicing in hospitals classified their cases by replacing “verbal analysis of subjectively defined sensations and feelings” with physical examination and observable organic structures.^29^ Case records are here the product of the hospital. They were systematic and quantifiable. In Jewson’s narrative, the place of the patient shifts as the medical cosmology changes. Bedside medicine produces narratives about individuals; hospital medicine produces cases, which constitute collections of observable data. Jewson’s definition of medical case was shaped by the hospital medicine of eighteenth-century Paris. This is the arena that produced the objective, natural body problematized by Barbara Duden in her study of women’s bodies through the printed observations of Johann Storch in eighteenth-century Germany. She argues, “The reality of this ‘body’ was the product of these descriptions [medical observations], and not vice versa, for what took hold was the belief that these clinical descriptions truly grasped and reproduced ‘reality.’”^30^ Jewson and Duden are responding to Michel Foucault’s trope about the clinical gaze and the creation of the medical subject.^31^ With a turn to the patient, historians of medicine began to consider medical narratives by patients and practitioners [Other P-604] and the conventions of medical record keeping.^32^ Casebooks became key sources for histories of medical encounters, but few scholars succeeded in identifying sources as rich as Napier’s casebooks and Storch’s observations, or bringing the methodological mastery of MacDonald or Duden to bear on them.^33^

To make best use of casebooks, we need to understand that they are historical artifacts as well as documents of past practices. Conventions for recording casebooks drew on changing forms of writing practices across a range of social, practical, and disciplinary arenas. The English casebooks with known provenance were written by practitioners ranging from literate artisans to university-trained physicians, partaking in widespread changes in writing practices that began in the sixteenth century with the increased availability of paper and shifting forms of literacy and scholarship.^34^ They seem to have been modeled on or are at least analogous to three sorts of writing: diaries, registers, and testimonials. Before considering these, it is instructive to compare the modes in which astrologers and physicians produced written records.

Astrological and medical casebooks are both serial records of practice. Conceptually, astrology and medicine were predictive arts, founded in the reading of signs, whether celestial or somatic.^35^ Astrology was taught within the medical curriculum in European universities from the thirteenth century.^36^ Astronomy, astrology, and medicine are credited with having [Other P-605] provided the foundations for the rise of observation as an epistemic genre.^37^ Learned medicine is useful for orienting the practices of Forman, Napier, and their contemporaries, but many English medical practitioners were not educated in the universities. Moreover, the use of horary astrology was generally frowned upon by learned physicians.^38^ Horary charts, based on the moment the question was asked, were sketched during the consultation. All forms of astrology required written computations. Astrologers needed to work on paper, while medical practitioners did not.

This distinction between astrological and medical records brings into focus the place of writing in early modern medical practices. Like the astrologers’ casebooks, medical diaries or journals were a first record of practice. Written during, or shortly after, a medical consultation, they were ordered by date with the patient’s name, the disease, the treatment, and occasionally a payment. The authors of medical diaries set out the headings and margins of the page appropriate to their needs. Like a shop book, a diary could fulfill its purpose simply by being written, providing a record of practice. Many medical diaries, like other forms of early modern writing, make sense within the model of textual transmission borrowed from financial bookkeeping. As merchants kept wastebooks transferred into ledgers and scholars kept memoranda digested into commonplaces, so medical practitioners kept diaries extracted into collections of recipes or commonplace books ordered by body part or disease. Often these were termed observations.^39^ The term “casebook” encompasses all of these forms of medical writing.

Administrative registers provided a second model for casebooks, often recorded in tall, narrow volumes, half the width of a folio page. This format was often used for keeping lists, which included registers, accounts, and inventories. Less concretely, the literary practices of ships’ officers probably shaped the habits of ships’ doctors. John Woodall’s 1617 instructions for surgeons include regular record keeping, and the records of [Other P-606] John Conney in the 1660s and William Cockburn in the 1690s provide evidence that such casebooks were written.^40^ From the 1660s, registers were kept of the people who received the royal touch for the king’s evil. These lists ensured that no person was touched more than once, and were later printed as evidence of the efficacy of the practice.^41^ However, there is little direct evidence for the influence of administrative registers on the record-keeping habits of specific practitioners.^42^

Finally, some casebooks seem to have been written as evidence of sound practices or successful cures, perhaps prompted by the dangers or practicing chymical medicine or modeled on Italian cure testimonials. These were produced by folk healers to advertise their services and justify their practices to the medical authorities. They may have contributed to the increasing practice of collecting cases, which testified to a practitioner’s expertise, often in instances where the effectiveness of the remedy could not be justified on doctrinal grounds.^43^ English examples include John Clarke’s 1602 pamphlet advertising the names of people he and other chymists had cured over the preceding two decades. As Clarke notes, printing “publike observations” of one’s cures was standard practice among Europe’s learned physicians and England’s producers of special oils and waters.^44^ In the 1630s another irregular London practitioner, John Evans, printed his cures alongside customer testimonials to advertise an antimonial cup that imparted a gentle purging effect to wine.^45^ The force of written cases against accusations of malpractice also motivated eminent physicians to keep casebooks.[Other P-607]

If casebooks can be like diaries, registers, or testimonials, what is excluded from the definition? It is in part negative, and the presence of medical cases amid other sorts of notes confirms the fluidity of early modern record-keeping practices. A casebook is not a collection of medical information, such as that made by Nathaniel Johnston, the Yorkshire physician, who kept individual case notes with extracts from medical works and recipes circa 1670 to 1690 in what falls under the loose heading commonplace book.^46^ Nor are commonplace books that record information about diseases and treatments.^47^ Medical curiosities—recovery from gunshot and dog bite—are also discounted.^48^ So too are collections of recipes, even if they name the people for whom they were prescribed, though the prescriptions books on which they are based might be included. These forms of writing, along with collections of astrological genitures and nativities, are cognate to the casebooks.^49^ Many of them are useful in discerning who kept medical records and for what purpose.

Having defined casebooks as serial records of practice, and situated them in terms of developments in learned medicine and paper technologies, the following sections consider the records themselves, taking astrological and medical casebooks in turn. Attention to the records focuses our attention on the various working processes that produced them.

## Astrological Records

I examined eleven sets of astrological records dating from the mid-fifteenth to the late seventeenth centuries. Richard Trewythian’s astrological casebooks are the earliest, and are probably representative of others that are lost.^50^ He recorded several dozen calculations from 1442 to 1458, noting them in the margins of his astronomical tables as well as in a separate notebook that also contained occasional notes about medical practice, book dealing, and money lending.^51^ From the fifteenth century, astrologers became increasingly reflexive about their records. What had begun [Other P-608] as instruments of analysis came to be seen as records of practice. In the Holy Roman Empire in the 1540s, Johannes Schöner collected notes of astrological events from four generations of scholars, posthumously published in the *Nachlass observationes*.^52^ In 1555, Thomas Bodier, a French astrologer and physician, published fifty-five decumbitures from the previous decade.^53^ Back in England, in the 1570s an anonymous astrologer recorded twenty pages of nativities and horary questions.^54^

Nothing prepares us to find Forman recording eighteen hundred consultations a year in the 1590s. His manuscripts suggest he began practicing in the 1580s and kept regular records from the early 1590s.^55^ In 1597 Forman began to teach Napier, who used Forman’s methods as the basis of his record-keeping habits for the next three decades.^56^ The occasional contribution by assistants is a reminder that early modern record keeping was often a collective endeavor. Napier relied especially on his curate and medical assistant, Gerence James, who contributes to Napier’s casebooks and may have kept his own records.^57^ Napier’s practice was continued by his nephew Sir Richard Napier (1607–76) at least through to the 1660s.^58^ Other evidence from the early seventeenth century is scarce. Two anonymous volumes record astrological cases, in much smaller numbers, from the 1610s and 1620s.^59^ William Bredon, who held a living in a Buckinghamshire parish from 1616 to 1638, had a busy astrological practice, but his records do not survive.^60^ In the 1630s Jeffrey Le Neve (1579–1653), after a failed career as a merchant and politician in Great Yarmouth, reinvented himself as an astrologer and moved to London. His casebooks are lost, but he extracted records of five hundred consultations from 1635 to 1641 into “Vindicta astrologiae judiciariae, or, The Vindication of Judiciarie Astrologie,” indexed by client’s name and question.^61^[Other P-609]

The traffic across the pages of Forman’s and Napier’s casebooks is matched by records from the 1640s. John Booker’s casebooks from 1648 to 1665 contain a thousand cases a year, systematically written in shorthand.^62^ The records of William Lilly, the most famous English astrologer in history, survive in an incomplete run from 1644 to 1666. Although unsystematic, at their peak, they record roughly two thousand cases a year.^63^ In portraits, like other astrologers, Lilly is represented writing notes on loose sheets. As physicians handed out prescriptions, so astrologers may have presented their clients with astrological charts. Like Lilly’s, the papers of Francis Bernard, an eminent Restoration apothecary and physician, record the sparse details of astrological cases, mostly genitures, with little order except the sequence of the consultations. He kept records of patients and prescriptions in separate notebooks.^64^ The records of Nicholas Culpeper do not survive, and less is known about the record-keeping practices of the dozens of other seventeenth-century London astrologers.^65^ In short, Forman’s casebooks mark a turning point in the tradition, and his and Napier’s records are more systematic and extensive than anything else that survives.

We would expect to find evidence about missing casebooks in the numerous astrological manuals printed in the second half of the seventeenth century.^66^ Lilly includes a few examples in his astrological manual, *Christian Astrology* (1647). Culpeper’s manuals are similarly lacking in cases.^67^ William Salmon’s medical works are rich with observations, while [Other P-610] his astrological works present general rules without particular examples.^68^ Astrologers kept written records, but they seldom drew examples from them to adorn their books.

By the middle of the century, astrologers began to adopt the language of observation and testimony, sometimes to teach or testify to good practice. Richard Saunders, an established London astrologer from the 1650s, set out detailed astrological rules without reference to cases, then appended a section of “experiments”^69^ drawn from “diligent observation” as “this Art is better taught by practice than speculation.”^70^ Joseph Blagrave, an astrologer–physician in Reading, included only several examples from his 1671 astrological manual, noting that he “inserted in this Book, the Names, and Places of dwelling of sundry Persons who have been by me cured of such Infirmities and Griefs aforesaid, and how performed; that so others may be informed how to do the like.”^71^

The irony surrounding astrological casebooks from the 1650s is that Elias Ashmole collected and preserved the records of previous generations of practitioners, while most of the papers of his contemporaries, willfully or not, perished.

## Medical Records

Physicians, in theory, carried the authority of literate knowledge in their robes and ended the day at a writing table. Pen and paper featured in their consultations when they wrote prescriptions or jotted memoranda. As the extant casebooks attest, various sorts of medical practitioners used a range of documenting practices.

The most extensive surviving medical casebooks are those of the famous Huguenot and Royal physician, Theodore de Mayerne. He filled more than three thousand pages with elaborate narratives of roughly a thousand cases from 1603 to 1653 (probably half his total practice), written in beautiful script with drawings of trusses, wigs, and syringes in the margins.^72^ Mayerne initially called his records “observationes medicinae,” [Other P-611] then in 1603 began a more digested volume of “ephemerides,” that is, day-by-day records, highlighting the value of medical theory.^73^ Fashioned as learned works, these documents may have had a more pragmatic impetus. Mayerne’s habits were formed in Paris during the antimony wars, and like other chymical practitioners he may have begun keeping records to guard against accusations of malpractice.^74^

At the other end of the spectrum are fragments of manuscripts containing a handful of cases on scraps of paper by unknown practitioners.^75^ Most extant casebooks range from pocket books to large folios, record a few hundred cases, in either or both Latin and English, with some spanning months, others years. Almanacs, which often served as diaries, were also used to record medical records.^76^ Often the hand is messy and the pages worn. Others, like Mayerne’s, were later collected into neat notebooks. The most comprehensive are headed with a name, date, and complaint, then list a history, diagnosis, remedy/therapy, and, in limited examples, a payment; the simplest list a name, disease, date, or remedy.^77^ Whether formatted like a diary or account book, written during a consultation or at the end of the day, or destined to be digested into observations or testimonials, these records share common features. They are all artifacts produced by the medical encounter.

Treating casebooks as found objects highlights how medical practitioners used writing as a tool for marking details or constructing narratives. Throughout the seventeenth century, English practitioners occasionally reflected on their record-keeping practices. Around 1600 Dr. Barker of Shrewsbury instructed the aspiring physician to “Note the patients name, day, houer, conditions of urin, disease, accidents, methal [i.e., mettle, meaning spirit], medicine, diet, government.” The format of the notebooks, he concludes, should vary depending on the case: “greate long cures note in folio / shorter common cures that come or send in half syde or quarter / note visiting cures in a manuell [i.e., pocket book].”^78^ Barker’s casebooks are missing, but a fragment headed “Observations & [Other P-612] cases in physic” shows him later digesting systematic records of the name, date, complaint, history, and remedy for a number of cases.^79^

In 1617 John Woodall, the surgeon and proponent of chymical medicine, recommended that the best way to learn the art of surgery was to observe “the whole passages of the diseased people”; this included the instruction to “keepe a Jornall in writing of the daily passages of the voyage in that kinde,” “of the unsuccessive applications, as of the successive.”^80^ Woodall encouraged students of surgery, like the Italian and Parisian students of physic in the previous century, to keep casebooks to aid their understanding of medicine.^81^

With the recovery and translation of Hippocratic texts in the sixteenth century, the ancient doctor became a model physician, recording the case and cure.^82^ In 1654, Culpeper translated a work by the German medical reformer Simeon Partlicius, which included an elaboration on ideas attributed to Hippocrates about the duties of a physician. Partlicius’s list of the doctor’s duties concludes with guidelines on how to order practice, overlapping with Woodall’s advice to surgeons. He should keep a “Catalogue of Authors,” a “Diary,” a list of notable “observations,” an herb garden, and a record of “his best Experiments in such an order that he may know redily how to find them.” Each day should begin with remembering what he did the day before. In the afternoon he should gather simples, study medical books, and visit his patients. In the evening he should reflect on what he did during the day, perhaps updating his diary, and “commit something to memory.”^83^ This is the same method that an eighteenth-century editor ascribed to the author of a set of seventeenth-century records. These followed an “indigested Method” and were probably a “sort of Diary in which the Author might set down things for his own Remembrance, when he return’d from visiting his Patients.”^84^ Memory and paper technologies, equally necessary for the assiduous scholar, enabled a practitioner to document a consultation once he had left the bedside.^85^[Other P-613]

Thomas Willis kept casebooks from 1650 to 1652, while he was participating in a circle of experimental philosophy at Oxford and working as a physician.^86^ He traveled to see his patients, and his one surviving notebook narrates fifty cases in a rushed Latin, probably, as the ideal scholar was advised, written at the end of the day and updated as the case progressed. Later Willis reflected that he wrote these notes as part of a Baconian program of study. Not finding the truths he was seeking in books, he “resolved with myself, to search into living and breathing Examples: and therefore sitting oftentimes by the Sick, I was wont carefully to search out their Cases, to weigh all the symptoms, and to put them, with exact Diaries of the Diseases, into writing; then diligently to meditate on these, and to compare some with others; and then began to adopt general Notions from particular Events. … ”^87^ This was the practice of compiling a medical commonplace book.

In the 1670s, the Dorchester-based physician Thomas Burwell, later to become president of the Royal College of Physicians, wrote an anonymous work against a competing German physician, Friedrich Loss, who had implicated Burwell, badly, in one of his cases. Loss had published a collection of Latin observations based on his practice. Burwell sets out, against Loss, that the patient and what ails him are the sole subjects that concern the physician. Burwell writes, “It hath been a custom with me, especially in Patients or Diseases of more than ordinary remark, to keep a Diary of my Practice, partly for my Patients sake, that I may the better understand what should be done for them, or what at any time I have done that they found good in; partly for my own sake, that I might have the surer foundation to build my experience upon in Physic.”^88^ Also in the 1670s, the elderly Norfolk physician Sir Thomas Browne wrote to his son, Edward, “You did well to sett downe in your booke a kind of diarie of your practice; tis good providence so to doe, and it may bee usefull hereafter unto you.”^89^

Barker, Woodall, Culpeper, Willis, Burwell, and Browne shared a presumption that writing was a useful tool for medical practice and implied that records served a later purpose. Such records were not necessarily [Other P-614] intended to endure. Recipes and cures recorded on erasable writing tablets by definition did not.^90^ These practitioners nonetheless differed in their methods and intentions. Barker stresses that the book should be specific to the cure. For Woodall, records are didactic, and tied to the study of books of surgery and physic.^91^ Culpeper, borrowing the voices of Partlicius and Hippocrates, emphasized that good medical practice was founded on notebooks, memory, and reason. Willis, at least in retrospect, saw his casebooks as part of a program of experimental philosophy. Burwell used his casebooks as a defensive record, kept initially for the benefit of the patient and in pursuit of reasoned medicine, later as a testament of good practice. Browne reminds us that early modern record keeping marked God’s will.

Guided by these doctors, the remainder of this article charts the history of English casebooks. Within this narrative, all known casebooks, whether the manuscripts survive or their existence has been inferred from other sources, are analyzed according to three overlapping contexts: production, retention and reuse, and collection and printing. In terms of production, casebooks were modeled on scholarly practices of keeping diaries, commonplaces and observations, testimonials, or administrative registers. Diaries and observations dominate, often borrowing attributes from testimonials and registers. Placing each casebook requires attention to the level of learning of the author, format of the paper, layout of the page, ordering of the records, and whether the script is rough or fair. The retention and reuse of casebooks, and the motives for these practices, makes sense within the changing medical politics of the seventeenth century, especially in relation to the disputes about chymical physic and the influence of the new philosophy. Finally, the collection and printing of casebooks provides evidence for the creation of the medical record as a class of historical documents. Often the records resist this regime.

The earliest English casebooks are akin to diaries and account books. John Crophill, an Essex bailiff and medical practitioner, recorded the names of patients and treatments among his account books in the late fifteenth century.^92^ This is not a series of medical cases, but it is a starting point for considering English casebooks. Then in the 1560s, a Scottish physician kept a remedy book listing the names of around 150 patients,^93^ [Other P-615] and a physician in Hampshire recorded cases amid recipes, accounts, and other miscellaneous notes from circa 1565 to 1573.^94^ Edward Barlow, an apothecary repeatedly accused of practicing without a license by the London College of Physicians, recorded lists of patients and prescriptions from 1589 to 1590.^95^ These are the decades when Forman was fashioning himself as an astrologer–physician and beginning to keep casebooks. By 1600, English medical practitioners were keeping casebooks in the forms of diaries and accounts.

I have associated diaries and account books as they are daily records of events or transactions, and were often kept together. Payments, however, are seldom found in casebooks, probably because practitioners wrote this information in separate books. Fewer than 3 percent of Forman’s cases and 25 percent of Napier’s cases record payments, while most medical casebooks record none.^96^ There are three exceptions. Elizabeth Thompson, the Kendal midwife, is the first. Midwives usually recorded payment, but Thompson included details of her cases.^97^ The experimental philosopher and physician Henry Power kept notes on his practice from 1665 to 1667, recording the patient’s name, date, and disease and charges for each remedy, with lists of accounts and recipes at the front.^98^ Finally, an anonymous practitioner recorded notes on alchemical texts and chymical recipes at one end of a notebook and listed further remedies and particular treatments from 1669 to 1674 at the other. Costs are included and the entries are crossed through, presumably to mark the payment of the debt.^99^ Once a debt was paid, there was probably little incentive for practitioners to retain evidence of fees.

Most of the extant English casebooks are diaries written by practitioners who fashioned themselves as physicians, regardless of their level of formal learning. Despite Woodall’s advice, limited numbers of surgical records survive. These, moreover, have little material difference from other casebooks, and are best treated alongside them. The exception is the casebook of Joseph Binns, the London surgeon whose practice has [Other P-616] been studied by Lucinda Beier.^100^ These evidence a haphazard system of record keeping with little evident scholarly imperative. Binns recorded 671 cases, probably only a sample of his practice, between 1633 and 1663.^101^ He wrote most of his cases on long, narrow pages, or pages folded into narrow columns. Other papers, such as the note written in the margin of a printed work and torn out or pages that have been folded in a pocket, were probably wedged into the original notebook.^102^ These papers are now bound into a large folio, leaving little indication of how Binns kept them. They include a date and sometimes a name at the top of the entry. Some are indexed by disease.^103^

Elizabeth Thompson probably did not model her records on those of physicians. Nor did Hugh Platt, the London improver and entrepreneur, or George Hill, an unlicensed apothecary. Platt’s casebooks are missing, but starting in 1605 he digested them into a book of cures, organized from “aches” to “ytch,” and listed remedies and examples of successful cures under each heading.^104^ In the late 1620s Hill similarly digested his casebooks into a book of “Experiments & Cures.” He headed each case with its disease, followed by the patient’s name, a narrative of the illness, and the cure.^105^ Like cure testimonials from the previous century, Platt and Hill used their casebooks as the basis for collections of cures that worked.

The remaining casebooks were kept by people who fashioned themselves as physicians. From 1592 to 1607 the London physician Stephen Bredwell (or Bradwell), son-in-law to the eminent surgeon John Banister, kept a “Diarium practicum” in a tall, narrow notebook, recording the patient’s name, complaint, and remedy, interspersed with recipes from other physicians.^106^ Thomas Marwood, a physician, recorded date, name, disease, and prescriptions for a few dozen cases from October 1635.^107^ A small chronological notebook records names, dates, and recipes for several hundred cases from 1636 to 1663, drawing a line under each case—a diary writing practice.^108^ An unknown physician working in London circa [Other P-617] 1638 to 1643 and Lancashire and Chester 1651 to 1662 kept notes of names, dates, sometimes a disease and copious quantities of prescribed medicines.^109^

John Pratt, a physician at Trinity College, Cambridge, recorded cases and observations from 1646 to 1661. Like Bredwell, he used a tall, narrow ledger, devoting one page to each case, detailing the patient’s name and date at the top, with the state of the disease, diagnosis, cure, and prescription listed below. Organized by date, the working nature of these records is confirmed by Pratt’s addition of a patient index at the front.^110^ William Petty, the Oxford professor of anatomy and later political arithmetician, recorded lists of prescriptions with names and dates, and sixty-seven “Observationes medicae et praxis,” perhaps based on casebooks.^111^ From 1655 to 1659 an anonymous London practitioner kept a compact chronological notebook in Latin. Patient names and sometimes addresses are noted in the left margin, with the recipe in the body of the text; dates are recorded at the end of some of the entries and, again, a line is drawn below each case.^112^ In New England in the middle decades of the century, John Winthrop Jr., the esteemed social leader and advocate of chymical physic, kept records of his busy medical practice, typically noting in brief the patient’s name, disease, and remedy.^113^ Finally, as already noted, the ship’s doctor John Conney wrote a “Diarium practicum” of six hundred remedies that he prescribed while at sea circa 1661 to 1664.^114^

One of the common features of these casebooks is their inclusion of remedies. Further scrutiny of particular sets of records could provide evidence about whether physicians dispensed much medicine and little advice, as their opponents complained. Likewise, more work needs to be done on whether physicians sold their own preparations. What is clear is that remedies feature prominently in casebooks. Perhaps this was pragmatic. Recipes had long served to store and transmit bundles of empirical knowledge.^115^ It is possible that the prescription of a remedy contributed to the likelihood that a case would be recorded. In some instances, a physician’s [Other P-618] prescriptions survive without associated case records. John Downes kept prescriptions written on long pieces of paper along with other medical and devotional notes in the 1670s.^116^ The “diarie of your practice” for which Sir Thomas Browne praised his son Edward survives in the form of chronological notes of patients’ names and the remedies he prescribed for them from 1675 to 1678. These may have complemented casebooks written over a longer period.^117^ Manuscripts containing recipes may also have been retained when others were discarded.^118^

The value of recording remedies is further evidenced in the collections of cases by named doctors made by third parties. Prescriptions and details of cures were collected as testaments to good practice. For instance, in Cambridge circa 1625 to 1628 an anonymous scribe collected around a hundred cases, mostly by John Gostlin, an established university physician, documenting his disputes with John Nichols. The records note practitioner, patient, an occasional date, symptoms, remedy, and the success of the cure. The scribe favored Goslin’s treatments, and perhaps he was the apothecary who filled these prescriptions.^119^ In a rare example of surviving physicians’ prescriptions, Jeremiah Webbe, an Oxford apothecary, collected the original scripts for dozens of receipts in 1653.^120^

Remedies similarly feature in more systematically ordered casebooks. An anonymous notebook from circa 1605 to 1611 records cases of cures, many effected through chymical remedies, alongside recipes and observations.^121^ John Symcotts, a rural physician, selected records of eighty-three cases from his decades of practice in Huntingdonshire, Bedfordshire, and Cambridgeshire, evidencing his collection of medical recipes from numerous sources.^122^ Percival Willughby, the Derbyshire physician and man-midwife, kept records, now missing, of cases from the 1630s through [Other P-619] the 1670s, which informed his “Observations on childbirth.”^123^ Dr. Bate neatly collected roughly two hundred cases from 1654 to 1659 into an alphabetical notebook organized by name with the date in the left margin and prescriptions following.^124^

There are examples of disease-centered collections. In the 1610s a practitioner collected several dozen cases alongside gynecological commonplaces.^125^ More elaborately, John Hall, the Stratford physician and Shakespeare’s son-in-law, assembled a chronological collection of 182 cases from 1611 to 1635, probably based on rough notes.^126^ The first forty-six record name, age, date, disease, and prescription, then the format changes and each entry begins with a disease, followed by details of the case and then the name, age, and date. An index of diseases is included at the end of the volume. This collection is written in Latin and modeled on the *observationes* of learned physicians. Similarly, in 1652–53 the Northampton physician John Metford methodically recorded “Observationes & Curationes,” heading each entry with the disease and a number, and digesting these into an index at the end. He also recorded copious remedies, correlating them to diseases.^127^

Chymical practitioners continued to keep casebooks. The anonymous practitioner who recorded payments, wrote notes on alchemical texts and chymical recipes at one end of a notebook, with the other listing further remedies and particular treatments from 1669 to 1674.^128^ One Dr. Bellingham maintained an interest in alchemical texts and remedies, recording ten cases under “some observations of the working of some medicines” in 1679.^129^

The contest between Galenic and chymical schools of medicine came to a head in this period, and cases featured throughout their printed debates. William Walwyn, the leveler and lay physician who promoted Helmontian medicine, must have kept written records detailing the age, sex, disease, and remedy of his patients. His *Healths New Store-House Opened* (1661) [Other P-620] and *Physick for Families* (1669) recount dozens of cases demonstrating the effectiveness of his mild remedies.^130^ Thomas O’Dowde, the Royalist physician who attempted to establish a society of chymical physicians to rival the College of Physicians, used cases to promote the virtues of chymical physic.^131^ George Thompson’s *Aimatiasis: Or, the True Way of Preserving the Bloud* (1670) denounced Willis’s promotion of bloodletting, using a single, elaborate case, taken from “among many I have taken notice of.” Thompson, unlike O’Dowde, built his argument for chymical physic from reason, not cases.^132^

Cases played a related but distinct role in the inquiries of the nascent experimental philosophers. John Ward, an Oxford-trained physician and clergyman, famously wrote sixteen small diaries from 1648 to 1681 documenting natural philosophical and medical inquiries in Oxford and London. These are chronological commonplace books, and the volumes from 1658 to 1669 include cases from his medical practice interspersed with historical and physiological speculations often drawn from other authors.^133^ John Locke similarly recorded medical cases in his journals during the 1660s,^134^ while Locke’s collaborator, Thomas Sydenham, recorded the cases that informed his histories of diseases in *Observationes medicae circa morborum acutorum historiam et curationem* (1676).^135^ Thomas Wharton, at St. Thomas’s Hospital, London, recorded names, addresses, diseases, and prescriptions for around 150 cases in his 1663 almanac, indexed by disease, perhaps in preparation for a junto on histories of diseases at the College of Physicians.^136^ As already noted, Henry Power’s records of practice from 1665 to 1667 include the patient’s name, date, disease, and charges for each remedy.^137^ These are the years when Loss and Burwell, the Dorchester physicians, kept records of their competing practices.

Despite the ructions over chymical physic and the advent of experimental philosophy, the practices of writing casebooks remained unchanged [Other P-621] throughout the century, yet fewer diaries and more collections of observations survive from the final decades. An exception is John Merewether’s use, much like Thomas Wharton’s two decades earlier, of an almanac as a medical diary. Merewether, a Wiltshire physician, recorded his “Praxis medica” beside monies and books lent, borrowed, and received on pages interleaved in *Riders British Merlin* that he bought annually for a decade from 1688.^138^ From 1676 to 1696 Sir Edmund King, the surgeon, physician, and experimental philosopher, kept a book of cases listing date, name, age, disease, remedy, and the occasional outcome (“she dyed”).^139^ Casebooks are included among the dozens of volumes written by Christopher Love Morley, a Catholic physician, educated in Leiden and practicing in England in the 1680s. One volume of his notes is written in a uniform format, headed by a date on the left of the page, name in the center, and disease on the right, with details of the case and remedy below. The other volume presents the remedies in a tabular form. Both volumes are roughly chronological and seem to be complementary.^140^

Printed books based on casebooks fared well in the second half of the seventeenth century. The surgeon Richard Wiseman used now lost casebooks as the basis of hundreds of cures in *A Treatise of Wounds* (1672).^141^ The young Hans Sloane recorded observations of his medical practice from the 1680s.^142^ As already noted, William Cockburn, a ship’s doctor in the early 1690s, kept a journal of his practice that was the basis for his book about the diseases of seamen.^143^ This was probably the norm among medical members of the maritime community, but most of the evidence for these practices dates from later decades.

Two changes are evident in attitudes toward cases and casebooks in the second half of the seventeenth century. Observations in their pure form were a scholarly genre to advance knowledge, yet the label “cases” [Other P-622] was increasingly used more broadly, and included printed medical cases that served the dual purpose of advertising expertise and establishing credit. Daniel Turner, the eminent London surgeon, inverted the genre in his *Apologia Chyrurgica: A Vindication of the Noble Art of Chyrurgery* (1695), which calls for the regulation of practice in light of a series of cases in which quacks harm their patients by telling “many stories of their grand Achievements.” Turner believed “these mens Credit is built on such lying and romantick stories.”^144^ By the end of the century, including cures in broadsides and pamphlets was commonplace as practitioners capitalized on London’s medical marketplace.^145^ Learned practitioners became wary of the use of cases to testify to skill, but did not stop including them in their books.

Second, cases and casebooks became objects of collection. As Ashmole began collecting astrological casebooks in the 1650s, medical cases and casebooks were increasingly collected and occasionally printed. Hall and his practice were famous, in part because he was married to Shakespeare’s daughter, and James Cooke, a surgeon, translated, edited, and augmented the work for publication in 1657, with subsequent editions in 1679 and 1683. The astrologer William Salmon kept records of his practice from the 1670s and digested hundreds of his and other practitioners’ cases in published observations, promising thousands more. Published in weekly installments from July 1681, each focused on a disease and set out the constitution of the sick body, the symptoms, causes, method of cure, and composition of remedy. These are based on his practice, with choice observations from notable physicians such as Mattheaus Platerius, Martin Ruland, Thomas Willis, and Abraham Zacutus interjected. Salmon’s manuscripts are lost, but he saw print as a form of perpetuity and included in his observations “cases which have fallen into our Hands in *Manuscripts*, the which by Reason of the Service they may do the publick, We are Unwilling should Perish by the Devouring Jaws of Time.”^146^ Others were similarly motivated to print what had become historical casebooks. Excerpts from Mayerne’s casebooks were published as *Praxis Mayerniana* in two volumes (1690, 1696), followed by another compilation, *Mayernii Opera* [Other P-623] *Medica*, in 1700. In 1715 the anonymously published *General Observations and Prescriptions in the Practice of Physick* brought to light the casebooks of Daniel Oxenbridge, a physician who worked in London from the 1620s.^147^ Sloane had a different approach to the preserving casebooks. In the early decades of the eighteenth century, as this article attests, he collected the majority of English examples that are now extant.

## Conclusion

This article began with an astrological consultation, the astrologer seated at his table, pen and paper at the ready. Through a comparison of astrological and medical records across two centuries, it charted developments in the ways in which English practitioners kept records and reflected on their practices. Astrologers had a long history of working from particular moments, stellar configurations, and events to general rules. These practices required systematic notation. Physicians increasingly modeled themselves on Hippocrates, recording details of cases as the basis for reasoned expositions of the histories of disease. Medical records, as other scholars have demonstrated, shaped the production of medical knowledge. The nature of these records as artifacts of medical encounters has instead been the focus of this article. Medical and astrological casebooks were serial records of practice. The term encompasses diaries, the observations into which they were digested, collections of successful cures, and registers of patients, remedies, and diseases. Casebooks derived from multiple traditions and a plurality of motives, converging in imperatives to write things down, which became increasingly prevalent across the spectrum of literate medical practitioners during the seventeenth century. Practitioners explored different methods of recording cases, using them to produce improved medical knowledge, to advertise sound methods, and to document the history of past practices. Forman’s and Napier’s casebooks are unique, but not unusual. Early modern medical records were produced within local medical politics and the broader worlds of paper technologies and epistemic genres. Casebooks document medical practices, but they also shaped them. The processes of producing the records—from jotted notes to printed observations—are as important to the history of medicine as the final product.[Other P-624]

## Footnotes

1

MeltonJohn Astrologaster, or the Figure-Caster 1621), 89. For the sake of brevity, the following conventions are applied in the notes. Manuscript references are in a short form. Early modern printed books have been located with the English Short Title Catalogue and often read on Early English Books Online. Where I have drawn on a person’s entry in the online edition of the Oxford Dictionary of National Biography (http://www.oxforddnb.com), I refer to ODNB.

2

BeierLucinda McCray Sufferers and Healers: The Experience of Illness in Seventeenth-Century England (London Routledge 1987 ChurchillWendy Female Patients in Early Modern Britain: Gender, Diagnosis, and Treatment (Farnham Ashgate 2012 DudenBarbara The Woman Beneath the Skin: A Doctor’s Patients in Eighteenth-Century Germany, trans. DunlapThomas 1987 Cambridge, Mass Harvard University Press 1991 KassellLauren Medicine and Magic in Elizabethan London: Simon Forman, Astrologer, Alchemist, and Physician (Oxford Oxford University Press 2005 MacDonaldMichael Mystical Bedlam: Madness, Anxiety, and Healing in Seventeenth-Century England (Cambridge Cambridge University Press 1981 NanceBrian The Art of Medical Portraiture: Turquet de Mayerne as Baroque Physician (Amsterdam Rodopi 2001 SawyerRonald "Patients, Healers and Disease in the Southeast Midlands, 1597–1634" Ph.D. diss University of Wisconsin 1986 For the use of letters, diaries, etc., see BresadolaMarco "A Physician and a Man of Science: Patients, Physicians and Diseases in Marcello Malpighi’s Medical Practice," Bull. Hist. Med 85 2011 193221 PorterDorothyPorterRoy Patient’s Progress: Doctors and Doctoring in Eighteenth-Century England (Cambridge Polity 1989 SmithLisa Wynne "Secrets of Place: The Medical Casebooks of Vivant-Augustin Ganiare," in Secrets and Knowledge in Medicine and Science, 1500–1800, ed. LeongElaineRankinAlisha (Farnham Ashgate 2011), 21331; StolbergMichael Experiencing Illness and the Sick Body in Early Modern Europe, trans. UnglaubLeonhardKennedyLogan 2003, Basingstoke Palgrave 2011 WeisserOlivia "Boils, Pushes and Wheals: Reading Bumps on the Body in Early Modern England," Soc. Hist. Med 22 2009 32139. On the place of the patient in medical history, see ArmstrongDavid "The Patient’s View," Soc. Sci. Med 18 1984 73744; CondrauFlurin "The Patient’s View Meets the Clinical Gaze," Soc. Hist. Med 20 2007 52540; PorterRoy "The Patient’s View: Doing Medical History from Below," Theory Soc 14 1985 17598; Roy Porter, "The Patient in England, c.1660–c.1800," in Medicine in Society, ed. WearAndrew (Cambridge Cambridge University Press 1992), 91118. For the use of casebooks from other periods, see DavisGayle "The Cruel Madness of Love": Sex, Syphilis and Psychiatry in Scotland, 1880–1930 (Amsterdam Rodopi 2008 StoweSteven M. Doctoring the South: Southern Physicians and Everyday Medicine in the Mid-nineteenth Century (Chapel Hill University of North Carolina Press 2004 SturdySteve "Knowing Cases: Biomedicine in Edinburgh, 1887–1920," Soc. Stud. Sci 37 2007 65989.

3
The term comes from Anke te Heesen, "The Notebook. A Paper Technology," in Making Things Public: Atmospheres of Democracy, ed. LatourBrunoWeibelPeter (Cambridge, Mass. MIT Press 2005), 58289.

4
See SmythAdam Autobiography in Early Modern England (Cambridge Cambridge University Press 2010 The laboratory notebooks of Gershom Bulkeley, StarkeyGeorgeVaughanThomas would also reward being read as examples of early modern autobiographical writing: WoodwardWalter W. Prospero’s America: John Winthrop, Jr., Alchemy and the Creation of New England Culture, 1606–1676 (Chapel Hill University of North Carolina Press 2010), 243.

5

BlairAnn M. Too Much to Know: Managing Scholarly Information before the Modern Age (New Haven, Conn Yale University Press, 2010 BlairAnnYeoRichard eds., "Note-Taking in Early Modern Europe," special issue of Intellectual History Review20 2010 DyerChristopher A Country Merchant, 1495–1520: Trading and Farming at the End of the Middle Ages (Oxford University of Oxford Press 2012 Müller-WilleStaffanCharmantierIsabelle "Natural History and Information Overload: The Case of Linnaeus," Stud. Hist. Philos. Biol. Biomed. Sci 43 2012 415; OgbornMiles Indian Ink: Script and Print in the Making of the English East India Company (Chicago University of Chicago Press 2007 ShermanWilliam H. Used Books: Marking Readers in Renaissance England (Philadelphia University of Pennsylvania Press 2008 HeesenAnke te "Accounting for the Natural World: Double-entry Bookkeeping in the Field," in Colonial Botany: Science, Commerce, and Politics in the Early Modern World, ed. SchiebingerLondaSwanClaudia (Philadelphia University of Pennsylvania Press 2004), 23751; VineAngus "Commercial Commonplacing: Francis Bacon, the Waste-Book, and the Ledger," in "Manuscript Miscellanies c.1450–1700," ed. BeadleRichardBealPeterBurrowColinEdwardsA. S. G. Engl. Manuscript Stud. 1100–170016 2011 197218.

6

KassellLaurenHawkinsMichaelRalleyRobertYoungJohn eds., A Digital Edition of Simon Forman’s and Richard Napier’s Medical Records, 1596–1634, http://www.magicandmedi-cine.hps.cam.ac.uk. On Forman, see also KassellLauren "How to Read Simon Forman’s Casebooks: Medicine, Astrology and Gender in Elizabethan London," Soc. Hist. Med 12 1999 318; Kassell Medicine and Magic (n. 2); RowseA. L. Simon Forman: Sex and Society in Shakespeare’s Age (London Weidenfeld & Nicolson 1974 TraisterBarbara H. "Medicine and Astrology in Elizabethan England: The Case of Simon Forman," Trans. Stud. Coll. Phys. Philadelphia2 1989 27997; Traister, The Notorious Astrological Physician of London: Works and Days of Simon Forman (Chicago University of Chicago Press 2001 On Napier, see Mac-Donald, Mystical Bedlam (n. 2); Sawyer, "Patients, Healers and Disease" (n. 2).

7
On physicians’ complaints that women consulted irregular practitioners, see PellingMargaret "Compromised by Gender: The Role of the Male Medical Practitioner in Early Modern England," in The Task of Healing: Medicine, Religion and Gender in the Netherlands, 1450–1800, ed. MarlandHilaryPellingMargaret (Rotterdam Erasmus 1996), 10133.

8

ThomasKeith Religion and the Decline of Magic: Studies in Popular Beliefs in Sixteenth- and Seventeenth-Century England 1971, Harmondsworth Penguin 1973 MacDonald, Mystical Bedlam (n. 2); Sawyer, "Patients, Healers and Disease" (n. 2). See also Kassell Medicine and Magic (n. 2), 12730.

9

Kassell "How to Read Simon Forman’s Casebooks" (n. 6); Kassell Medicine and Magic (n. 2). Cf. Andrew Wear, Knowledge and Practice in English Medicine, 1550–1680 (Cambridge Cambridge University Press 2000), esp. 12652 41256 on physicians’ authority at the bedside, their use of illness narratives, and competing models of disease.

10
I inherited this term from RowseA. L. . For a discussion of the medical use of the word "case," see ForresterJohn "If p, Then What? Thinking in Cases," Hist. Hum. Sci 9 1996 125.

11
For work that draws on English casebooks into the eighteenth century, see ChurchillWendy "The Medical Practice of the Sexed Body: Women, Men, and Disease in Britain, circa 1600–1740," Soc. Hist. Med 18 2005 322; Churchill Female Patients (n. 2).1598138010.1093/sochis/hki006

12

DrageWilliam A Physical Nosonomy 1664), ODNB.

13 His notebooks are Sloane MSS 1626, 1627, 1628, 1629, 1636, 1644, 1646, 1647, 1658, ODNB. For medical cases, see Sloane MSS 1636, fol. 32; 1644, fols. 29v, 57v; 1658, fol. 14v.

14 Sloane MSS 1087, 1602, ODNB.

15
On this in relation to medieval medicine, see JonesPeter Murray "Witnesses to Medieval Medical Practice in the Harley Collection," eBLJ 2008), article 8, http://www.bl.uk/eblj/2008articles/article8.html.

16

SiraisiNancy History, Medicine, and the Traditions of Renaissance Learning (Ann Arbor University of Michigan Press 2007), chap. 2.

17

PomataGianna "Praxis Historialis: The Uses of Historia in Early Modern Medicine," in Historia: Empiricism and Erudition in Early Modern Europe, ed. PomataGiannaSiraisiNancy (Cambridge, Mass MIT Press 2005), 10546 esp. 12236. See also AgrimiJoleCriscianiChiara Les Consilia Médicaux, trans. ViolaCaroline Typologie des sources du Moyen Age occidental, no. 69. Institut d’Etudes Médiévals, Université Catholique de Louvain (Turnhout, Belgium Brepols 1994 CriscianiChiara "Histories, Stories, Exempla, and Anecdotes: Michele Savonarola from Latin to Vernacular," in Pomata and Siraisi Historia, 297324 esp. 30911; JonesPeter Murray "Consilium—Narratio—Memorandum: Types of Medieval Case History" (unplublished typescript); PomataGianna "Sharing Cases: The Observationes in Early Modern Medicine," Early Sci. Med 15 2010 193236. Surgical works also featured cases: McVaughMichael "Treatment of Hernia in the Later Middle Ages: Surgical Correction and Social Construction," in Medicine from the Black Death to the French Disease, ed. FrenchRoger  (Aldershot Ashgate 1998), 13155; NuttonVivian "Humanist Surgery," in The Medical Renaissance of the Sixteenth Century, ed. WearAndrewFrenchRogerLonieI M (Cambridge Cambridge University Press 1985), 7599.

18

ParkKatharine "Observation in the Margins, 500–1500,"1544 and PomataGianna "Observation Rising: Birth of an Epistemic Genre, 1500–1650,"4580 esp. 49, both in Histories of Scientific Observation, ed. DastonLorraineLunbeckElizabeth (Chicago Chicago University Press 2011

19

SiraisiNancy "Historiae, Natural History, Roman Antiquity, and some Roman Physicians," in Pomata and Siraisi, Historia (n. 17), 32554 esp. 341.

20
Pomata, "Praxis Historialis" (n. 17), 129.

21
Pomata, "Observation Rising, (n. 18), 5466 esp. 57; Pomata, "Praxis Historialis" (n. 17), 131, 134; Pomata, "Sharing Cases" (n. 18), 20811.

22

BaconFrancis The Advancement of Learning 1605), book 2.X.4; cf. Pomata, "Praxis Historialis" (n. 17), 129.

23
On this stereotype of Sydenham, see AckerknechtErwin A Short History of Medicine 1955, Baltimore Johns Hopkins University Press 1982), 122.

24

HessVolkerMendelsohnJ. Andrew "Case and Series: Medical Knowledge and Paper Technology, 1600–1900," Hist. Sci 48 2010 287314.

25
[ TerneChristopher ], Some Papers Writ in the Year 1664 1670), 27 On the politics surrounding this work, see CookeHarold J. The Decline of the Old Medical Regime in Stuart London (Ithaca, N.Y Cornell University Press 1986), 14143.

26
[ Terne ], Some Papers (n. 25), 31.

27
For this phrase, and a model of careful attention to a casebook as an artifact, see RenziSilvia De "A Career in Manuscripts: Genres and Purposes of a Physician’s Writing in Rome, 1600–1630," Italian Stud 66 2011 23448.10.1179/174861811X13009843386639PMC326866722298938

28

JewsonNicholas "The Disappearance of the Sick-Man from Medical Cosmology, 1770–1870," Sociology10 1976 22544 quotation on 228, reprinted in Internat. J. Epidemiol 38 2009 62233 with commentaries on its significance for medical sociology and the history of medicine.

29 Ibid., 229–30.

30

Duden Woman Beneath the Skin (n. 2), 4.

31

FoucaultMichel Birth of the Clinic: An Archaeology of Medical Perception, trans. SheridanA. M. 1963, London Tavistock Press 1973

32

FissellMary "The Disappearance of the Patient’s Narrative and the Invention of Hospital Medicine," in British Medicine in an Age of Reform, ed. WearAndrewFrenchRoger (London Routledge 1991), 92109; Porter "Patient’s View" (n. 2); Porter "Patient in England" (n. 2); RisseGuenter B.WarnerJohn Harley "Reconstructing Clinical Activities: Patient Records in Medical History," Soc. Hist. Med 5 1992 183205; WarnerJohn Harley "The Uses of Patient Records by Historians—Patterns, Possibilities and Perplexities," Health Hist 1 1999 10111.

33 See note 2 above.

34 See note 5 above.

35

BroeckeSteven vanden "Evidence and Conjecture in Cardano’s Horoscope Collections," in Horoscopes and Public Spheres: Essays on the History of Astrology, ed. OestmannGüntherRutkinH. DarrelStuckradKocku von (Berlin W. de Gruyter 2005), 20724; HackingIan The Emergence of Probability: A Philosophical Study of Early Ideas about Probability, Induction and Statistical Inference, 2nd ed. (Cambridge Cambridge University Press 2006), xiiixiv; MacleanIan Logic, Signs and Nature in the Renaissance: The Case of Learned Medicine (Cambridge Cambridge University Press 2001), 33536; Park "Observation in the Margins" (n. 18); WestmanRobert The Copernican Question: Prognostication, Skepticism, and the Celestial Order (Berkeley University of California Press 2011

36

HaytonDarin The Astrologers of Emperor Maximilian I: Nature, Knowledge, and Politics in the Holy Roman Empire (Pittsburgh University of Pittsburgh Press forthcoming); Hayton, "Instruments and Demonstrations in the Astrological Curriculum: Evidence from the University of Vienna, 1500–1530," Stud. Hist. Philos. Biol. Biomed. Sci 41 2010 12534; RutkinH. Darrel "Astrology," in The Cambridge History of Science, Volume 3, Early Modern Science, ed. ParkKatharineDastonLorraine (Cambridge Cambridge University Press 2006), 54161; SiraisiNancy Medieval and Early Renaissance Medicine (Chicago University of Chicago Press 1990

37

Park "Observation in the Margins" (n. 18).

38

GraftonAnthony Cardano’s Cosmos: The Worlds and Works of a Renaissance Astrologer (Cambridge, Mass Harvard University Press 1999 GraftonSiraisiNancy "Between the Election and My Hopes: Girolamo Cardano and Medical Astrology," in Secrets of Nature: Astrology and Alchemy in Early Modern Europe, ed. NewmanWilliamGraftonAnthony (Cambridge, Mass MIT Press 2001), 69131.

39
On the influence of mercantile practices ca. 1600, see esp. Smyth Autobiography (n. 4); Vine, "Commercial Commonplacing" (n. 5).

40

WoodallJohn The Surgions Mate 1617), sig. ¶¶3v. Conney’s records are Sloane MSS 2766, fols. 232; 2779. CockburnWilliam An Account of the Nature, Causes, Symptoms, and Cure of the Distempers That Are Incident to Seafaring People 1696), ODNB. For a few pages from the casebooks by a ship’s doctor, perhaps ChambarsGeorge in the 1660s, see Sloane MS 379, fols. 3539. On Cockburn, see CookHarold "Practical Medicine and the British Armed Forces after the ‘Glorious Revolution,’" Med. Hist 34 1990 126.10.1017/s0025727300050249PMC10359982405217

41

BrowneJohn Adenochoiradelogia, or an Antomick-Chirurgical Treastise of Glandules and Strumaes 1684), bk. III, 8588.

42
On registers kept by learned societies, see Vine, "Commercial Commonplacing" (n. 5), 210.

43

PomataGianna Contracting a Cure: Patients, Healers and the Law in Early Modern Bologna (Baltimore Johns Hopkins University Press 1998), 5052; Pomata, "Observation Rising" (n. 18), 12627; Pomata, "Praxis Historialis" (n. 17), 12526; Pomata, "Sharing Cases" (n. 17), 213 See also EamonWilliam Science and the Secrets of Nature: Books of Secrets in Medieval and Early Modern Culture (Princeton Princeton University Press 1994

44
44 ClarkeJohn The Trumpet of Apollo Sounding Out the Sweete Blast of Recoverie, in Diverse Dangerous and Desperate Diseases 1602), sigs. A34.

45

EvansJohn The Universall Medicine: Or the Virtues of My Magneticall or Antimoniall Cup 1642 The 1634 edition of this work was destroyed by order of the College of Physicians.

46 Wellcome MS 3083.

47 Royal College of Physicians, MS Cole 221.

48 Sloane MS 3169. This seems to have been written by one John Spence or Spencer in the 1640s and 1650s.

49
On geniture collections, see GraftonAnthony "Geniture Collections, Origins and Uses of a Genre," in Books and the Sciences in History, ed. Frasca-SpadaMarinaJardineNick (Cambridge Cambridge University Press 2000), 4968. Astrologers whose detailed life writings survive include FormanSimonJeakeSamuel of Rye. See also the astrological diary of PurslowNorris a Wapping clothier, ca. 1678–1703: Wellcome MS 4021.

50
See, for instance, Thomas Religion and the Decline of Magic (n. 8), 357n.81.

51
Sloane MS 428; PageSophie "Richard Trewythian and the Uses of Astrology in Late Medieval England," J. Warburg Courtauld Inst 64 2001 193228; Thomas Religion and the Decline of Magic (n. 8), 357.

52
Pomata, "Observation Rising" (n. 18), 49.

53 Ibid., 54.

54 Uncatalogued manuscript, University of Illinois Library, Urbana-Champaign.

55

Kassell Medicine and Magic (n. 2), 133, 136 (on his record keeping).

56

KassellLaurenHawkinsMichaelRalleyRobertYoungJohn "List of the manuscripts of Forman’s and Napier’s casebooks," Casebooks Project, http://www.magicand-medicine.hps.cam.ac.uk/the-manuscripts/manuscripts-list (accessed September 7, 2014).

57

Sawyer "Patients, Healers and Disease" (n. 2), 13738.

58 Sir Richard’s records are bound, and in some cases shared, with his uncle’s: Ashmole MSS 174, 204, 240, 416, 412, 211. The survival of his records after his uncle’s death is patchy: Ashmole MSS 122, 177, 421, 421, 1388, ODNB.

59 Ashmole MS 336, item 1, is a book of about three hundred horary figures dating from 1611. Ashmole MS 388 is a collection of horary figures, arranged thematically, from May 15, 1614, to June 13, 1619. These records are continued in Ashmole MS 336, item 2, through 1620. Ashmole MS 349, item 2, fols. 1–70 is “The Booke of Judicialls for Severall Astrological Questions,” a collection of cases on lost and stolen goods and marriage prospects ca. 1614.

60

Sawyer "Patients, Healers and Disease" (n. 2), 143.

61 Ashmole MS 418. For a corrupt Latin version of the text, see Ashmole MS 400, ODNB.

62
For Booker’s papers, see BlackWilliam Henry A Descriptive, Analytical, and Critical Catalogue of the Manuscripts Bequeathed unto the University of Oxford by Elias Ashmole (Oxford, 1845) and MacrayW. D. Index to the Catalogue of the Manuscripts of Elias Ashmole (Oxford, 1866), ODNB; Thomas Religion and the Decline of Magic (n. 8), 363ff.

63
Ashmole MSS 184, 178, 185, 210, 420, 427, 430; Thomas Religion and the Decline of Magic (n. 8), 363ff.

64 Sloane MSS 91 (names of patients etc.), 1707 (charts), 1805 (recipes and prescriptions). These date from the 1680s. ODNB.

65
On Culpeper: ODNB; Thomas Religion and the Decline of Magic (n. 9), 36364. Other astrologers: CappBernard Astrology and the Popular Press: English Almanacs 1500–1800 (London Faber & Faber 1979 MacDonaldMichael , "The Career of Astrological Medicine in England," in Religio medici: Medicine and Religion in Seventeenth Century England, ed. GrellOle PeterCunninghamAndrew (Aldershot Scholar 1996), 6290; Thomas Religion and the Decline of Magic (n. 8), 35682. Sloane MS 2817 contains some long cases from the 1660s, but it is not a casebook.

66
For earlier examples, see FageJohn Speculum Aegrotorum. The Sicke-mens Glasse 1606), sig. E1. Similarly, G. C., A Treatise of Mathematicall Phisicke, appended to Claude Dariot, A Briefe and Most Easie Introduction to the Astrologicall Judgment of the Starres, trans. WitherFabian 1598), contains a single example: sig. [J4].

67
For instance, CulpeperNicholas Semeiotica Uranica: Or, an Astrological Judgment of Diseases from the Decumbiture of the Sick 1651), 40ff.

68
68 SalmonWilliam Synopsis Medicinae: Or a Compendium of Astrological, Galenical, & Chymical Physick 1671 Salmon Horæ mathematicæ, seu, urania: The Soul of Astrology 1679

69

SaundersRichard The Astrological Judgment and Practice of Physick 1677), 171ff. The work was published posthumously.

70 Ibid., 173.

71

BlagraveJoseph Blagraves Astrological Practice of Physick 1671), 84ff.

72
Sloane MSS 2058-76; Royal College of Physicians MS 444. See Nance Art of Medical Portraiture (n. 2), 36. For a notebook that contains records kept by Mayerne and his mentor, Jean de La Rivière, in Paris in the 1590s, see Sloane MS 2089.

73

Nance Art of Medical Portraiture (n. 2), 30, 51, 54.

74 Ibid., 54.

75 Sloane MS 739, fols. 10v–13, 57v, dated 1646–49; Sloane MS 810, Dr. Nathaniel Hodges’s medical observations, with a case and prescriptions on fol. 423ff; Sloane MS 3990 lists names, remedies, and a few details about the case in Latin in a tidy italic hand.

76
On almanacs as diaries, see KassellLauren "Almanacs and Prognostications," in The Oxford History of Popular Print Culture, Volume One: Cheap Print in Britain and Ireland to 1660, ed. RaymondJoad (Oxford Oxford University Press 2011), 43142.

77 For a rare example of a list of payments, see Sloane MS 2286, fols. 67v–68r, 70v, 71v.

78 Sloane MS 79, fol. 153. This appears amid his miscellaneous collection of medical notes.

79 Sloane MS 78, fols. 155–89.

80

Woodall Surgions Mate (n. 40), sig. ¶¶3v.

81 See note 21 above.

82

Siraisi "Historiae" (n. 19), 34.

83

PartliciusSimeon A New Method of Phisick, trans. CulpeperNicholas 1654), 107 This seems to be an extrapolation from pseudo-Hippocrates, "Decorum," Hippocrates, ed. JonesW. H. S. vol. 2 (London Heinemann 1923), 267301.

84 [Daniel Oxenbridge], *General Observations and Prescriptions in the Practice of Physick* (1715), sig. [A2v].

85

YeoRichard "Between Memory and Paperbooks: Baconianism and Natural History in Seventeenth-Century England," Hist. Sci 45 2007 146.

86
Wellcome MS 799A; DewhurstKenneth ed., Willis’s Oxford Casebook (1650–52) (Oxford Sandford 1981 Two other notebooks are missing: Dewhurst, 63.

87

WillisThomas Dr. Willis’s Practice of Physick Being All the Medical Works of That Renowned and Famous Physician, trans. PordageSamuel ([1659] 1681), 55.

88

MedicusAlius [ BurwellThomas ], Animadversions on the Medicinal Observations of … Mr. Frederick Loss 1674), 86; LossFriedrich Obersvationum medicinalium libri quatuor 1672

89

KeynesGeoffrey Works of Sir Thomas Browne, vol. 6 1931), 201, cited in MannSophie "Religion, Medicine, and Confessional Identity in Early Modern England" Ph.D. diss King’s College London, 2014), 89.

90

StallybrassPeterChartierRogerMoweryJ. FranklinWolfeHeather "Hamlet’s Tables and the Technologies of Writing in Renaissance England," Shakespeare Quart 55 2004 379419 esp. 403.

91

Woodall Surgions Mate (n. 40), sig. ¶¶4.

92 Harley MS 1735, pt. 2, fols. 36v–52v, ODNB.

93 Sloane MS 1197. Names are listed on fols. 1–2, 191–92.

94 British Library, Additional MS 28023.

95 Ashmole MS 1487.

96

KassellLaurenHawkinsMichaelRalleyRobertYoungJohn "Search results summary (Circumstances: Financial information)," Casebooks Project, http://www.magicandmedicine.hps.cam.ac.uk/the-casebooks/search/cases/results/summarise?ci=2 (accessed November 7, 2014).

97
Cumbria Record Office MS WD/Cr/1, cited in EvendenDoreen The Midwives of Seventeenth-Century London (Cambridge Cambridge University Press 2000), 128; on midwives’ account books, see 125, 12730.

98
Sloane MS 1351. See WilliamsKatherine E. "Hysteria in Seventeenth-Century Case Records and Unpublished Manuscripts," Hist. Psychiatry1 1990 383401.1161392310.1177/0957154X9000100402

99 Sloane MS 3773. For treatments/accounts, see fols. 92v–50v (from the back of the volume).

100
Sloane MS 153; Beier Sufferers and Healers (n. 3), 5196.

101 Ibid.

102 Sloane MS 153, fol. 153v.

103 Sloane MS 153, fol. 147.

104
Sloane MS 2209. On Platt, see HarknessDeborah The Jewel House: Elizabethan London and the Scientific Revolution (New Haven, Conn. Yale University Press 2007 MukherjeeAyesha "The Secrets of Sir Hugh Platt," in LeongRankin Secrets and Knowledge (n. 2), 6986.

105 Sloane MS 333, fols. 23–42.

106 Sloane MS 275; see also Sloane MS 461.

107 Royal College of Physicians, MS MARWT 440. Most of the names have been crossed through but are still legible.

108 Sloane MS 462.

109 Sloane MS 1055, fols. 31–113. Discussed in Williams, “Hysteria” (n. 99).

110 Sloane MS 80. Sloane MS 79, fols. 94–109v are longer cases written by Pratt. Sloane MS 58, fols. 1–18 is a pocket book containing four cases (fols. 1–18), then blank except some medical notes written from the other end.

111 B. L., Additional MS 72891, fols. 92–112 (prescriptions), 258–68 (observations). For the latter in English, see Additional MS 72892, fols. 10–21, printed in *The Petty Papers*, ed. Marquis of Lansdowne (London: Constable, 1927), 2:218–26.

112 Sloane MS 426. Many of the names are illegible due to damage. Cf. Sloane MS 462.

113

Woodward Prospero’s America (n. 4), 18499.

114 Sloane MS 2766, fols. 2–32. Sloane MS 2779 is Conney’s journal.

115

LeongElaine "Collecting Knowledge for the Family: Recipes, Gender and Practical Knowledge in the Early Modern English Household," Centaurus55 2013 85103; Pomata, "Sharing Cases" (n. 17), 206.10.1111/1600-0498.12019PMC370912123926360

116 Sloane MS 204.

117 Sloane MS 1865, fols. 62–98, ODNB.

118 Sloane MS 2595.

119 Sloane MS 1408, ODNB. William Butler’s recipes were collected by Mayerne and others: Sloane MSS 1991, 3746, fol. 30. For recipes attributed to Bredwell, see Sloane MS 108, fol. 59v.

120 Sloane MS 564. Most were by “Dr. Barksdale.”

121
Sloane MS 640. Remedies were also the focus of a Cambridge practitioner who collected eighty-two cases in a small notebook between 1619 and 1622: Sloane MS 1112; Beier Sufferers and Healers (n. 2), 12326; Beier "Seventeenth-Century English Surgery: The Casebook of Joseph Binns," in Medical Theory, Surgical Practice: Studies in the History of Surgery, ed. LawrenceChristopher (London Routledge 1992), 4884.

122

Beier Sufferers and Healers (n. 2), 99120 and passim. Symcotts’s records are augmented by surviving correspondence. PoynterF. N. L.BishopW. J. A Seventeenth-Century Doctor and His Patients: John Symcotts, 1592?–1662, Bedfordshire Historical Record Society vol. 31 (Streatley, Beds 1950

123

WillughbyPercival Observations in Midwifery (Warwick, 1863 Willughby’s notes toward this book are Sloane MS 529, fols. 119. See WilsonAdrian The Making of Man-Midwifery: Childbirth in England, 1660–1770 (Cambridge, Mass Harvard University Press 1995

124 Royal College of Physicians, MS 893.

125 Sloane MS 63.

126
London, British Library Egerton MS 2065; HallJohn Select Observations on English Bodies of Eminent Persons in Desperate Diseases 1657 LaneJoan John Hall and His Patients: The Medical Practice of Shakespeare’s Son-in-Law (Stratford-upon-Avon Shakespeare Birthplace Trust 1996

127 Sloane MS 2812.

128 Sloane MS 3773. For treatments/accounts, see fols. 92v–50v (from the back of the volume).

129 Sloane MS 630, fols. 158–62. Sloane MS 647 contains Bellingham’s correspondence relating to medical and chymical information.

130 Walwyn includes cases from Lazare Riviére’s observations at the end of *Physick for Families*.

131
O’DowdeThomasThe Poor Mans Physician1664O’DowdeThe Poor Man’s Physician1665

132

ThompsonGeorge Aimatiasis: Or, the True Way of Preserving the Bloud 1670), 9197.

133
Folger Shakespeare Library, MSS V.a.284-99, available online at luna.folger.edu. See FrankRobert G.jR. "The John Ward Diaries: Mirror of Seventeenth Century Science and Medicine," J. Hist. Med. & Allied Sci 49 1974 14779.459847910.1093/jhmas/xxix.2.147

134

MeynellG. G. "John Locke’s Method of Common-Placing as Seen in His Drafts and His Medical Notebooks," Seventeenth Cent 8 1993 24567.

135 ODNB.

136 Royal College of Physicians, MS WHART 641. The almanac is George Wharton, *Calendarium Carolinum* (1663). Almanacs owned by Wharton in subsequent years (MSS 642-44) do not contain medical cases.

137 Sloane MS 1351. See Williams, “Hysteria” (n. 98).

138
Wiltshire and SwindonRecord Office 2220/1/1-9, cited in Smyth, Autobiography (n. 4), 46 The virtuoso Martin Lister also recorded lists of patients and fees in almanacs in the 1670s: RoosAnna Marie Web of Nature: Martin Lister (1639–1712), the First Arachnologist (Leiden Brill 2011), 14548.

139 Sloane MS 1589, ODNB. King’s papers—Sloane MSS 1586–1598, 1640, 4038, 4078—deserve further study.

140 Sloane MSS 1256, 1258, ODNB. In the 1680s and 1690s John Kent, a surgeon, included observations from other authors and his own practice under headings for diseases followed by remedies and observed cases: Sloane MS 1527.

141
See SandassieSamantha "Evidenced-Based Medicine? Patient Case Studies in English Surgical Treatises, 1660–1700," Med. Human 34 2008), 1118 for the use of cases in other surgical works.10.1136/jmh.2008.00026623674534

142
SloaneHansA Voyage to the Islands Madera, Barbados, Nieves, S. Christophers and Jamaica1707

143

CockburnWilliam Account of the Nature (n. 40).

144

Turner Apologia Chyrurgica: A Vindication of the Noble Art of Chyrurgery 1695), 12.

145 See, for instance, *Jones of Hatton-Garden, His Book of Cures* (1673) and other works by George Jones.

146

SalmonWilliam Iatrica: Seu Praxis Medendi. The Practice of Curing Being a Medicinal History of above Three Thousand Famous Observations in the Cure of Diseases 1679), sig. [A1v]. See also Salmon, Parateremata: Or Select Physical and Chyrurgical Observations 1687) and his 1689 translation of Ysbrand Diemerbroeck’s The Anatomy of Human Bodies, to which are appended dozens of the Dutch physician’s observations and cures. ODNB.

147 ODNB.

